# No Trespassing! Abandoning the Novice/Expert Problem

**DOI:** 10.1007/s10670-024-00794-8

**Published:** 2024-03-08

**Authors:** Neil Levy

**Affiliations:** 1 Department of Philosophy, Macquarie University, Sydney, Australia; 2 Uehiro Centre for Practical Ethics, University of Oxford, Oxford, UK

## Abstract

The novice/expert problem is the problem of knowing which apparent expert to trust. Following Alvin Goldman’s lead, a number of philosophers have developed criteria that novices can use to distinguish more from less trustworthy experts. While the criteria the philosophers have identified are indeed useful in guiding expert choice, I argue, they can’t do the work that Goldman and his successors want from them: avoid a kind of testimonial scepticism. We can’t deploy them in the way needed to avoid such scepticism, because it would take genuine expertise to do so. I argue that attempts to deploy them in this sort of deep way involve a kind of transgression akin to, and at least as unreliable as, epistemic trespassing. We should give up trying to solve the novice/expert problem and instead promote better epistemic trust.

We rely on expert testimony for questions that are pressing for us: *Should I get the bivalent booster*? *Should I invest my money in crypto*? *Should I cut sugar from my diet*? *Should I vote for a candidate who promises to lower my income tax*? *Should I buy this beachfront property, ignoring predictions of sea level rise*? Very few of us have the data and the competence to answer even one of these questions for ourselves; that is, on the basis of our own assessment of the evidence. Instead, we’re forced to rely on experts. Experts are people who possess far greater capacities or far more evidence (or both) than non-experts in a particular domain of inquiry, and as a consequence are significantly more reliable at answering questions in that domain.^1^ We can’t acquire expertise in more than a very small number of domains, for want of time and (often) capacity. We’re therefore required to turn to the experts for advice.

All too often, however, the experts (or people who appear to be experts) offer conflicting advice. There are physicians and public health experts who urge us to get the bivalent booster, but there are many epidemiologists and physicians who disagree (see Griffin, 2021 for examples). Sometimes, dissent comes not from within a field, but from genuine experts in some *other* field, who argue that the field they criticize is unreliable on this issue. There are, notoriously, many examples from climate change denialism.

Novices like me therefore need some basis for deciding what to believe when apparent experts offer conflicting advice. Philosophers have risen to the challenge: they have offered a set of criteria we can apply to settle which expert is more likely to be reliable (Goldman, 2001; Anderson, 2011; Guerrero, 2017; Brennan, 2020; see De Cruz, 2020 for a somewhat more pessimistic view). These criteria are intended to be general: whenever we find ourselves faced with conflicting testimony from apparent experts, we can utilize these criteria to settle which expert is more reliable, and on that basis distinguish better advice from worse.

The stakes of this debate are high. We live in an age of hyperspecialisation (Millgram, 2015), where we are all pervasively dependent on experts to guide our decisions. We rely on doctors’ advice for our health, on mechanics for the maintenance and repair of our cars, on scientific advice to choose between policies, and so on and on. When advice conflicts, we often must make a choice between apparent experts, and our very lives may sometimes depend on choosing well.

In this paper, I will argue for pessimism about our capacity to utilize these criteria, or any successor criteria, to choose between conflicting experts *when the task is construed as these philosophers construe it*. We can and do use the criteria in settling who to trust, but we don’t and can’t use them in the way the philosophers ask us to. When genuine experts make confident judgments about controversies in fields beyond their own, they engage in what has come to be called *epistemic trespassing*. I will argue that the attempt to utilize the criteria in the way philosophers envisage amounts to an epistemic transgressionof an analogous sort. Epistemic trespassing results in judgments that are unreliable: it is, in the jargon, a defeater. The novice version of epistemic trespassing, which occurs when lay people use the criteria in the way they’re supposed to, is also a defeater for their judgments.

We can and do utilize the criteria the philosophers advance, but only to *extend our trust* from some sources, people, and judgments to new sources, people, and judgments. It follows, I will suggest, that the criteria are almost always unhelpful in just the kinds of situations where philosophers have (rightly) suggested we most need them: when we’re genuinely at a loss who to trust, or when we’re on the wrong side of an issue. In these circumstances, we don’t trust well enough for the criteria, as we can actually use them, to get a grip on the issues. Attempts to deploy them in such cases won’t reliably move us from doubt or unjustified belief to a better justified judgment.

## What Do Want from a Solution to the Novice/Expert Problem?

There are two broad ways of deciding who to believe when expert testimony conflicts – that is, of attempting to solve what Goldman (2001) calls the novice/expert problem. You can either attempt to assess the issue for yourself, or you can attempt instead to assess the reliability of the testifiers. Goldman and his successors have opted for the latter approach, and they’ve done so for very good reasons. As they recognize, the first approach is extremely unpromising. By hypothesis, experts have a significant edge on us in virtue of their training. Solving the novice/expert problem by settling the issues on which the (apparent) experts disagree will therefore require us to acquire a very significant slice of their expertise.^2^ We lack the skills and the evidence experts possess, and none of us can hope to acquire them in more than a *very* small number of domains. In this light, philosophers have instead advanced criteria that target the reliability of the experts rather than the reliability of their claims. We can’t settle debates between experts for ourselves, but we can hope to identify which of the contending experts is more likely to be reliable.

The criteria are intended to be domain-general, in the sense that they apply to (at least) most debates. Whenever apparent experts offer laypeople conflicting advice, we can use the criteria to decide between them. They’re also intended to be relatively easy to apply, requiring only the resources available to the average conscientious person. To some degree, indeed, they might seem quite commonsensical.

I will argue that the criteria Goldman and his successors advance are genuinely appropriate for distinguishing reliable from unreliable experts. As a matter of fact, we do deploy these criteria, or something like them, in making judgments between experts, and in doing so we may reasonably hope to come to possess knowledge. But Goldman and his successors aim for more than a descriptively adequate account of how people come to trust experts and thereby acquire knowledge (when they do). They want criteria that will *underwrite* the reliability of the resulting judgments. That is, they want these criteria to serve as a kind of backstop in justification – something to which we can point to make contrastive judgments: *this* novice is trusting well, whereas *that* one is trusting badly. To put it somewhat metaphorically, they see the criteria as *adding epistemic value* to novice judgment.

This desideratum for accounts of expert choice has gone unnoticed. Nevertheless, I think it’s reasonable to see it as a significant motivation for the accounts. The motivation is clearly on display in Goldman’s (2001) agenda-setting paper. There, he explicitly aims to avoid what he sees as the “testimonial scepticism” (86) inherent in John Hardwig’s (1991) approach to expert testimony. Hardwig accepts that novices can acquire knowledge from expert testimony. But in his view, they can’t really have good reasons for accepting such testimony. Instead, their trust is groundless.^3^ Though he doesn’t put it this way, in effect Hardwig is claiming that when we trust the right experts we thereby obtain knowledge, but we’re lucky to trust well. We lack good reasons for trusting one side of a debate rather than another.

Goldman, like Hardwig, is an externalist: that is, he holds that knowledge does not require that we have access to the basis on which our beliefs are justified. Nevertheless, he suggests we are not doomed to groundless trust: we can and should confer more or less trust in testimony depending on facts to which we do have access. He borrows here from debates over testimony in more everyday contexts. Reductionists about testimony hold that we must have positive grounds for accepting testimony; some evidence about the reliability of the person offering it, for example. Non-reductionists deny this. Goldman sees Hardwig as offering a non-reductionist account of a sort, according to which the sort of grounds that we might hope for to justify expert testimony are unavailable. Goldman denies this: he looks to the criteria to offer us evidence that will “often *bolster* or *defeat*” our justifiedness in accepting testimony (88). These criteria offer us grounds to distinguish good from bad testifiers. The criteria he advances add value in the following sense: they orient us to evidence about the source’s reliability or unreliability.

The hope that the criteria for expert choice can play this sort of role is also on display in Elizabeth Anderson’s equally classic paper on the apparent conflict between our pervasive reliance on expert testimony and democratic legitimacy (Anderson, 2011). In her view, this is a tension that can be dissolved, because citizens have the “second-order capacity” to distinguish between reliable and unreliable experts. It’s apparent that this is an epistemic value-adding capacity: it allows us to distinguish those experts who *are* reliable from those who merely advance views we might find congenial. Right now, she maintains, our trust in testimony is too often ill-grounded, but we can better ground it in assessments of the reliability of experts. Equally, one can see a similar (albeit much qualified) hope in Johnny Brennan’s (2020) suggestion that we ought to cultivate our metacognition: by turning the spotlight on ourselves, we can come to a better assessment of how we assess expert reliability, and thereby improve our capacity to choose well.

I agree (once more) that the criteria Goldman, Anderson, Brennan, and others advance are actually utilized in choosing experts. Expert choice – like responsiveness to testimony generally – is neither reflexive nor automatic (Sperber et al., 2010; Harris, 2012; Mascaro and Sperber 2009). It’s sensitive to features of context and testimony that indicate unreliability. To that extent, extreme testimonial scepticism is unwarranted. However, there’s no particular reason to think such sensitivity distinguishes the good case from the bad. While the criteria filter out some instances of unreliable testimony, disputes persist and people find themselves at a loss who to trust because bad cases are too often not filtered out. In these cases, there are no grounds for thinking that *we* deploy these criteria well and *they* deploy them badly, and therefore no prospect for making the contrastive judgment that Goldman and his successors hope for. The criteria don’t add epistemic value in the way Goldman hopes. They’re not ways of underwriting our trust; rather, they’re ways of extending it from some sources, people, and claims to new sources, people, and claims. It’s only when we *already* trust well that we can utilize the criteria to choose well.

## Criteria for Expert Choice

In his classic article, Goldman (2001) offered five criteria for identifying reliable experts: (1) evidence of dialectical superiority; (2) agreement from additional putative experts; (3) appraisal of the person’s expertise by ‘meta-experts’; (4) evidence of the experts’ interests and biases; and (5) evidence of the experts’ track records (93). These criteria have served as a template for later work on the novice-expert problem; they or their successors continue to be central to contemporary work. In this section, I will work through these criteria with the aim of showing that they can’t add value in the way Goldman and his heirs hope. I will argue that deploying them in a way that would genuinely add value – that is, amount to more than extending our trust in a source, person, or claim to a new source, person, or claim – would throw us back into making the very judgments the criteria are supposed to enable us to avoid, about matters beyond the capacity of non-experts to assess.

Criterion one turns on what Goldman calls dialectical superiority, which consists in a greater capacity to offer rebuttals and defeaters for rivals’ claims (Anderson also identifies this as a marker of reliability, under the heading “epistemic responsibility” (Anderson, 2011: 146). Allegedly, the novice can assess dialectical superiority even if they can’t assess the issues. But dialectical superiority allows us only to distinguish those with little familiarity with an issue, or those with little skill in argument, from the more skilled and the smoother. It’s of little use when we’re called upon to assess conflicting apparent experts. In cases like this (climate change debates offer plenty of examples), we can expect sophisticated debaters to offer smooth and superficially convincing arguments and rebuttals, whether they’re genuine experts or industry shills. Smooth and superficially convincing – that is, convincing to the layperson – rebuttals indicate familiarity with the issues and rhetorical prowess, not genuine expertise (Guerrero, 2017). In fact, we should expect well prepared debaters to outshine genuine experts every single time, unless the genuine experts are *also* skilled debaters. It’s very likely that disputes about climate change, among other issues, persist in part because both sides *appear* to offer good arguments and replies.

At first sight, agreement from other (putative) experts – Goldman’s second criterion –is far more helpful. First, it’s more easily assessed than dialectical superiority. The existence of a consensus on climate science (Cook et al., 2016) is, for example, widely reported in the media. Second, expert agreement is genuine evidence in favor of a view. This is the flipside of the epistemic significance of disagreement: Just as peer disagreement should lower my confidence by providing evidence that I may have made an error (Christensen, 2007; Matheson, 2015), so peer agreement provides evidence that I have *not* made an error. When an expert’s peers agree with her, our credence in her view should rise; other things being equal, a consensus or near consensus is a strong reason to accept expert testimony.

Moreover, this is a criterion that seems easily applied. Thanks to resources like Google Scholar, citation metrics are now widely available to the ordinary person. While citations are a crude measure of agreement (researchers may be cited for many reasons), citation counts are a good heuristic for esteem, and therefore are reasonably taken to indicate support either for a position (when the citations are to a particularly influential paper, for example) or for the expertise of the author (citations across the board).^4^

There are two problems with agreement from other experts as a criterion. Firstly, it risks simply pushing the bump in the rug to a new place. After all, dissenters on climate change can cite plenty of other (putative) experts who, they claim, agree with them. Assuming they’re honestly reporting support from other quarters, we’re forced into the position of assessing whether these other agents are, indeed, experts. I will set aside this issue, to focus on a different one. Dissenters often take the route not of denying that there’s a preponderance of opinion on the *other* side of the issue, but of arguing that the consensus did not arise in way that would make it reliable.

As Goldman emphasises, a preponderance of expert testimony adds weight to individual testimony only if the sources of testimony are independent of each other. The testimony of agents 1…n adds no weight to the credibility of an expert if these agents would concur with the expert no matter what she said. Goldman draws our attention to this sort of case not because he thinks that such reflexive deference is at all common, but because it highlights the role that independence plays in the evidential weight of testimony: we should discount testimony to the degree it’s not independent. Since consensus is, on the face of it, strong evidence in favor of an opinion, and consensus is relatively easily assessed, canny dissenters can be expected to focus on whether the consensus is sufficiently independent. And indeed, climate contrarians regularly accuse their opponents of ‘groupthink’: either of bowing to political pressure to say the ‘right’ things and concealing their private dissent, or of herding in their opinions due to non-rational pressures. Appealing to the weight of opinion doesn’t enable us to avoid hard questions; it forces us to confront them.

The task of assessing whether opinions are appropriately independent is much harder than usually thought, because we tend to overlook the extent to which dependence is *required* in expert domains. Science, the paradigm of such domain (or set of domains), is a deeply collective enterprise: every scientist is dependent on many other people, in multiple ways and in multiple contexts. A large majority of papers in most scientific disciplines are co-authored, with the average number of co-authors per paper rising steadily over the past century (Fanelli and Larivière 2016). Many papers are the product of hundreds, sometimes even thousands, of co-authors (Mallapaty, 2018). Work in a lab – and increasingly across labs – is distributed, with different researchers playing different roles. Typically, one co-author can’t replicate the work of another; it’s *always* the case that they must trust one another to carry out their work conscientiously and to report it accurately. Dependence extends beyond co-authors and lab members to the community as a whole. Scientists must take the opinions as well as the findings and theories of other scientists into consideration in calibrating their attitude to scientific claims, even in their own domains. For example, a biologist will be guided by the theory of evolution in her work even if she has no special expertise in evolutionary theory; she accepts that theory partly on the basis of trust, and would lower her confidence in some biological hypothesis were it (apparently) inconsistent with evolution. She likely dismisses whole research paradigms as dead ends, on the basis that others in her field have abandoned them. She’s not independent: she relies pervasively on others and they on her.

Dependence may sometimes be excessive, but assessing whether that’s the case is extremely difficult and requires detailed, field-specific, knowledge.^5^ Appropriate deference is domain-specific: computational linguists likely have patterns of appropriate deference that don’t resemble the patterns of appropriate deference exhibited by glaciologists; indeed, patterns of deference may differ markedly across subfields within a single discipline. It’s not insuperably difficult for an outsider to learn that psychologists sometimes defer appropriately to evolutionary theorists, or that neuroscientists should defer to the physicists involved in the refinement of fMRIs. But that’s nowhere near enough: it’ll require genuine domain-specific expertise to know what weight is appropriately placed on findings from other domains. Because we can rely on agreement from other experts only when it is appropriately independent, and we can’t assess whether opinion is appropriately independent without a good deal of genuine expertise, criterion 2 doesn’t give us the capacity to do an endrun around engaging with the evidence.^6^

A closely related worry applies with regard to citations. While citation counts are easily accessible and easily understood metrics, we might worry that they reflect inappropriate patterns of dependence. Perhaps dissenters aren’t cited not because their evidence is weaker or less persuasive, but because the in-group closes rank against the outgroup. Indeed, there’s every reason to think that citation patterns are heavily influenced by group opinions: scientists (for example) cite those researchers who are taken seriously in their community, and ignore the dissenters the community sees as cranks. Moreover, dissenting work will often have little visibility, since it is rarely published in high-profile journals. Mainstream scientists will insist that that’s because it is very rarely good enough to deserve such publication; dissenters will see it as the result of gatekeeping. To the extent that Carl Sagan’s adage that extraordinary claims require extraordinary evidence reflects scientific practice, we should expect that those who argue for a line that directly conflicts with what the mainstream holds will face more hurdles to publication.

Goldman’s third criterion is appraisal by meta-experts. Goldman says relatively little about it, apart from noting that credentials might be regarded as esteem by meta-experts. I’ll return to credentials soon. Beyond credentials, appraisal by meta-experts risks pushing the question up a level. How am I to assess which meta-experts – assuming there are such people – to trust? We seem to need a solution to the novice/expert problem – the problem the criterion is supposed to help us solve – to answer that question. In cases where there’s dispute about which expert to trust, we’re unlikely find agreement about who the reliable meta-experts are.

Perhaps we might rely on meta-analyses to distinguish more from less reliable experts. It is certainly true that meta-analyses have proven a useful resource, for example, for estimating effect sizes. However, they suffer from two problems. First, meta-analyses often suffer from a file drawer effect: since unsuccessful studies struggle to be published, they are much less likely to be included, and the effect size estimated is therefore often much greater than the true effect size (which may, indeed, not be significantly different than zero). If meta-analyses ever come to be widely relied on in public debate, I would expect sophisticated dissenters to make much of this fact, and of the replication crisis (Bausell, 2021) more generally. It is true that there are steps that can be taken to improve the reliability of meta-analyses, for example by estimating the size of the file drawer. But, second, sophisticated dissenters will claim that the studies that are included in the meta-analysis reflect patterns of gatekeeping and inappropriate dependence, and not the unbiased pursuit of truth.

Goldman’s fifth criterion focuses on the putative expert’s track record. Here he has in mind evidence of their past success within their domain of expertise, in particular their track record in *prediction* (rather than track record in the sense in which it is more commonly used; i.e., publications, grants, and prizes). He notes an immediate problem with applying this criterion: given how abstruse expert domains can be, how can novices assess the degree to which experts’ predictions have been successful? Goldman maintains that this problem is often surmountable: even when a domain is esoteric, the success of individual predictions may be easily observable. For instance, while I have little hope of understanding how a large language model actually functions, I can easily observe the difference between ChatGPT working as designed and ChatGPT breaking down (in the latter case, it produces obvious nonsense or nothing at all when it’s queried).

There are surely situations in which this heuristic is useful: we’re trying to choose between two mechanics, say, and one has a better record of repair than the other. Even in this sort of case, though, the heuristic offers only limited guidance. For one thing, success in automobile repair is much more easily assessed by non-experts than is success in maintenance. The fact that your new brake pads work well is evidence that your mechanic is competent at replacing them, but it’s not good evidence that they were correct that they needed replacing. One might hope to compare their performance to that of other mechanics (how often do your friends’ mechanics advise brake pad replacement?) but differences might reflect differences in driving conditions or styles across individuals, rather than nefarious practice. Being in a position to assess *that* requires serious work, which is beyond most of us (we’d need a wide sample of drivers randomized to mechanics for a start). In the kinds of cases Goldman and his successors have in mind, matters are very much more difficult: the markers of success themselves are disputed.

In these cases, apparent experts often argue either that their opponents’ claimed successes are fabrications, or that their successes are not relevant to the question at issue. Sometimes, these disputes reflect honest disagreement. Such disputes will be difficult – to say the least – for the novice to assess. Think of disputes over the efficacy of lockdowns as a response to COVID, with economists accusing epidemiologists of ignoring indirect consequences for public health. The issue is *hard*. On the one hand, lockdowns probably did prevent many deaths from COVID. On the other hand, they probably also resulted in a slowdown in economic activity and psychological harms associated with isolation. The magnitude of these benefits and harms – and there are certainly many others on both sides – is intrinsically difficult to assess. It remains disputed even today what the effects of the 2008 recession on mortality were. It’s been estimated to have resulted in at least 10,000 extra suicides in Europe and North America (Reeves et al., 2014) and more than 250,000 extra cancer-related deaths in OECD countries (Maruthappu et al., 2016). But there’s also evidence that all-cause mortality tends to *fall* during a recession (Ballester et al., 2019). The apparent conflict between these findings remains unresolved. Mortality is one of the *easiest* outcomes to measure. If experts can’t settle disputes like this one, even about the accuracy of *past* predictions, what hope do we have?^7^

Of course, some disputes arise *dis*honestly. The dispute over climate change is partially caused and sustained by ‘merchants of doubt’ (Oreskes & Conway, 2011), who knowingly aim at promoting a false narrative (Supran et al., 2023). Dishonesty does not make a dispute easier to resolve for novices, though, because the dishonest side work to *ensure* it’s a difficult task. For example, they obfuscate evidence of track record. Climate contrarians dispute the climatic record; they argue that it has been intentionally or unintentionally manipulated to support the warming narrative. *By design*, these assertions are difficult (if not impossible) for the novice to assess. It’s true that sometimes records are genuinely exoteric and easily understood. But for that very reason, disputes with these features don’t tend to persist. In a slogan, were the criterion useful, we’d already have used it by now – we usually already have.

The same sorts of issues arise with regard to the *relevance* of expertise to the question at hand. During the COVID pandemic, disputes about who was in a good position to advise on public policy were central to debates. Should epidemiologists take precedence over economists or even political scientists? Dissenters might argue that some experts can’t assess the new case, because there are too many extraneous pressures that distort their judgments, or that they erroneously conflate the new case with previous ones, missing the features that render it unique. Again, assessing these accusations will be beyond the novice.

Goldman’s final criterion concerns evidence of distorting interests and biases. We might, for example, dismiss or at least downgrade climate change sceptics on the basis that they have financial incentives to deceive (Oreskes and Conway devote considerable space to this issue). But the sceptics level precisely the same accusation at their opponents. We’re quick to dismiss such accusations, and it seems clear that financial incentives do not operate as directly for most climate scientists. Nevertheless, it’s not unreasonable to think that financial – and non-financial incentives – play similar sorts of roles on both sides (Guerrero, 2017). Many scientists are fully or partially on ‘soft money’; that is, their salary is fully or partly dependent on continuing to bring in grant money. Scientists are also motivated by prestige, and the rewards prestige brings. It’s impossible to understand the replication crisis without supposing that scientists are often motivated by considerations besides truth; the replication crisis arises, in important part, from scientists’ prioritising ways of gathering and analysing data that conduce to publishability at the expense of truth (Ritchie, 2020).

In response to the accusation that scientists are motivated to support the consensus by financial, prestige and conformity pressures, it’s common to point out that there would be enormous rewards for scientists who produced strong evidence against the consensus on climate change or evolution. That’s true, but it’s also not unreasonable to believe that social and financial pressures would make it difficult to come to possess such evidence (supposing there were any). Knock-down evidence is rare in science; for claims with the broad sweep of anthropogenic climate change and evolution by natural selection, it almost certainly doesn’t exist. A scientist could come to possess strong evidence against these hypotheses only as the result of a research program that spanned many years; very plausibly, it would also require the cooperation of many other laboratories and many other researchers (recall the extent to which scientists are dependent on one another). It would be difficult to launch and sustain such a research program in the face of existing financial and social pressures. Of course, I’m not claiming that as a matter of fact climate scientists or evolutionary biologists are subject to the same distorting pressures as the merchants of doubt of the Heartland Institute. Rather, I’m claiming that the novice who seriously wonders who she should trust will find it difficult or impossible to judge the issue on this basis.

As already mentioned, Goldman notes that experts’ credentials may provide evidence that they’re trustworthy. Anderson (2011) puts more emphasis on credentials, which she says are easily accessed by “a simple Google search” (150). She argues that the higher up what she calls the “hierarchy of expertise” (146) an expert is, the more weight we should give to their testimony. So, someone with an undergraduate degree is trumped by someone with an MA, who is trumped by a PhD, who is trumped by an active researcher, who is trumped by someone who is the recipient of major grants and awards, and so on. These criteria are indeed sometimes helpful, but in persisting disputes we find excellent credentials on both sides. The merchants of doubt found willing allies in figures like Frederick Seitz (who was President of the National Academy of Science), and COVID anti-vaxxers trumpet the credentials of Robert Malone, who styles himself the inventor of mRNA technology (Bartlett, 2021). The merchants produce parallel institutions and give parallel prizes (Oreskes & Conway, 2011). They (typically) don’t grant parallel degrees, but they don’t need to; there have always been those with genuine credentials and genuine expertise who are willing to lend the merchants their support.

Novices must trust experts if they are to come to hold justified beliefs about a range of very significant issues. They must trust experts because they can’t judge these questions for themselves: they lack the evidence and the capacity required to assess the issues directly. Goldman, and those he inspired, aim to develop criteria novices can use to assess expert reliability that do an endrun around our incapacity to assess the issues. They haven’t succeeded at this aim: when novices find themselves uncertain who to trust, these criteria won’t come to their aid. To apply each criterion, we need deep knowledge of the science and its structure – knowledge that is available only to experts.

## Epistemic Trespassing and the Novice/Expert Problem

In the previous section, I argued that the criteria Goldman and his successors develop don’t do the kind of work they ask of them. When we find ourselves uncertain who to trust, or want to check whether we’re right in trusting one side, the criteria won’t help. It’s not that we don’t in fact utilize these criteria in deciding who to trust; rather, it’s that they don’t confer the kind of justification that Goldman and his heirs hope for. I might, say, trust expert A over expert B because I think B is compromised by financial interests, but I don’t have the kind of expertise required to reliably assess whether A’s exposure to such interests is less compromising than B’s. In this section, I will go further: I will argue that attempts to apply the criteria in such cases amount to an epistemic transgression closely akin to epistemic trespassing.

A trustworthy expert is someone who testifies that *p* for epistemic reasons, and whose expertise qualifies them to assess whether *p*. One reason to discount expert testimony, therefore, is that the expert (however sincere, skilled, and well-motivated) lacks the domain-specific competence to assess whether *p*. Someone with the expertise reliably to assess claims about, say, the evolution of viruses will typically lack the competence to assess whether the benefits of lockdowns outweigh the costs. The genuine expert who adjudicates questions in a *different* expert domain on the basis of their own assessment (rather than on the basis of testimony) is an *epistemic trespasser* (Ballantyne, 2019b; DiPaolo, 2022; Gerken, 2023).^8^

Epistemic trespassers are (other things equal) unreliable sources of information, since the topic about which they testify is beyond their expert competence. Expertise is domain-specific, and an expert in one domain may be a novice in another, even when the domains are closely related. Ballantyne (2019a), whose discussion has set the agenda for discussion of the issue, suggests that sometimes trespassers can defend themselves on the grounds that their skills transfer to a new domain, and it is surely sometimes true that outsiders can make a significant contribution to a field. Sometimes they’re *more* reliable than the experts; perhaps the field is corrupt (for example). Epistemic trespassers are, however, typically far less reliable than domain experts, and in the absence of field-specific expertise may lack the competence to assess *whether* they’re reliable in the domain (recall that it takes domain-specific expertise to know to whom, and how much, to defer). In the absence of clear signs that the domain is corrupt (such as a bad track record for the domain as a whole, and not just for possibly unrepresentative individual researchers within it), we should regard the claims of epistemic trespassers as unreliable when they conflict with those of the domain experts.

Epistemic trespassers are, by definition, genuine experts. Despite being experts, their claims are suspect. But if *they’re* not reliable outside their domain, what hope do we non-experts have? If we should be very reluctant to believe that experts are able to adjudicate issues outside their own domain, then we should be even more sceptical that novices can adjudicate issues in expert domains. I want to suggest that for the novice, choosing between contending experts in persisting disputes just *is* adjudicating an issue outside their domain of competence. Attempts to solve the novice/expert problem are not themselves instances of epistemic trespassing. That’s true by definition: epistemic trespassing is defined as a transgression committed by an expert, and a novice is not an expert.^9^ But they have all the features that make epistemic trespassing unreliable.^10^ The ‘no trespassing’ sign applies to novices and outside experts alike. Let’s call the novice version of the tort *epistemic trespassing*_*n*_.

Working through Goldman’s criteria for identifying reliable expertise as we did in the previous section reveals the extent to which deploying them requires epistemic trespassing_n_. We saw how assessing the dialectical superiority of one apparent expert over another requires genuine understanding of issues, if it is not simply to be driven by differences in rhetorical prowess. We saw how assessing the weight of apparent agreement requires judging the extent to which experts are appropriately independent of one another, and that that requires deep knowledge of the domain. Similarly, assessing the extent to which incentives distort debates requires expertise, as does assessing the content and the relevance of experts’ track record. In each case, the novice who makes a judgment on these issues adjudicates a question in an expert domain, and such adjudication is epistemic trespassing_n_. Even assessing whether one of the contending experts is an epistemic trespasser, or whether their trespassing is excused, *itself* involves epistemic trespassing_n_; it requires making judgements about the bounds of domains and about the extent to which skills in one domain transfer to another.^11^

When dispute arises honestly, asking the novice to assess which side is better justified calls upon them to adjudicate a dispute that *by hypothesis* even experts in that domain find difficult. Since the experts can’t agree on the question at issue, what hope does the novice have? For disputes that arise dishonestly, the novice is called upon to adjudicate questions about the structure of a domain, and therefore whether the deference patterns it manifests are appropriate, or between interpretations of data one of which has been cleverly designed to appear convincing, or to assess whether an expert is an epistemic trespasser and, if they are, whether their skills transfer to the new domain. Every single one of these questions requires expertise: either expertise in the domain to which the first-order issue belongs (e.g., is climate change caused by anthropogenic carbon emissions?) or meta-level expertise – assuming there is such a thing – in how domains relate to one another. Novices cannot hope reliably to address them.

Social epistemologists have followed Ballantyne’s lead in identifying epistemic trespassing as a defeater: we should regard the judgments of an epistemic trespasser as (at least) prima facie unreliable. In this section, I’ve argued that in putting forward criteria for expert choice, we have in effect been advising novices to engage in epistemic trespassing_n_. But epistemic trespassing_n_ is the novice version of epistemic trespassing, and at least as unreliable as the expert version. In offering this guidance, we’re encouraging irresponsible and unreliable epistemic behavior.

## Trusting the Expert

It’s important to be clear that I am *not* claiming that novices don’t use the criteria that Goldman and his successors identify to choose between (apparent) experts. Rather, I’m claiming that novices don’t and can’t deploy the criteria in the kind of value-adding way that Goldman calls for. Goldman hopes that his criteria can allow us to avoid the kind of testimonial scepticism that threatens if we say, with Hardwig, that novice trust in experts is groundless. He’s wrong: the criteria don’t underwrite the contrastive judgment he hopes for. We do deploy the criteria, but this is true on all sides. It’s not that *we* are sensitive to expert credentials (for example), and *they* are not. Rather, we are sensitive to the credentials issued or endorsed by the institutions and people *we* trust, and they are sensitive to the credentials issued or endorsed by institutions and people *they* trust. For the most part, we already deploy the criteria, as well as we can be expected to. We can’t deploy them better because it would take expertise to do so.

We actually deploy the criteria to extend our trust, rather than in a way that could ground it. Because we already trust mainstream institutions (well-known universities, national and international scientific academies, the mainstream media, or what have you), we tend to trust claims and people they endorse. Our use of credentials is a way of extending our trust from *these* degree-conferring institutions, or *those* prize awarding institutions, to *these* experts or *those* judgments. Our use of consensus is a way of extending our trust: from *these* scientific societies or *these* well-known journals to *those* claims. We discount testimony from an apparent expert when we regard the source of their funding as potentially corrupting; here we extend our *dis*trust. It’s often the case, though, that our assessment of the funding source as a source of bias is itself at least partly grounded in testimony from trusted sources: canny merchants of doubt ensure that their money isn’t obviously corrupting (as O’Connor and Weatherall (2019) highlight, the tobacco industry spent a great deal of money funding research, with the aim of cultivating friendly experts as well as highlighting real, but very rare, causes of lung cancer other than smoking). It takes expertise to see how this funding was corrupting in the way that NIH funding is not.

Similarly, our assessments of track record are based on trust: we trust the testimony of other experts that the research reported is reliable, and not fabricated as the dissenters claim. We trust that the methods of gathering and analysing data were appropriate, and that the apparent evidence reported by sceptics is cherry picked (at best). We’re not assessing these things for ourselves. We can’t. Nor can we engage in the deeper inquiry required to show that the consensus we rely on is appropriately formed or that the funding sources we see as innocuous do not introduce undue distortions. If worries like these must be addressed before we’re able to rely on testimony from those experts we trust, then we will never be in a good position to be able to rely on such testimony. Conversely, if we can indeed – as all sides agree – come to know how things stand in expert domains on the basis of testimony, then we don’t need to be able to address such worries.

Extending our trust in this sort of way might reasonably be seen as adding some epistemic value, in just the same way that epistemic vigilance (Sperber et al., 2010) – our sensitivity to evidence of the competence and trustworthiness of those who offer us testimony – adds epistemic value. Our trust in testimony isn’t groundless. But it doesn’t add the sort or magnitude of value that Goldman hopes for from it. It ensures that our trust is extended from old cases to new cases. It doesn’t avoid the need for trusting; rather, it’s founded on it. Nor does it issue in a contrastive judgment; not, at least, in typical cases. When one side of a dispute – say climate change sceptics – trusts badly, it’s not typically because they have failed to check the credentials, the track record, or the funding sources of the apparent experts they defer to.^12^ It’s because they extend their trust from unreliable sources, institutions, and people to the new case. Deploying the criteria, however conscientiously, wouldn’t help.

Reminders of the criteria are sometimes helpful: sometimes, it’s useful to be prompted to check the credentials of apparent experts, whether they have a financial interest in the advice they’re offering, whether they’re supported by other experts or represent a small minority, and so on. But unless we already trust well – in the credential-granting institutions, for example, or in the scientific journals – these reminders won’t help us. They won’t ground our trust, they help us extend it. ‘Trust well’ may not be very useful advice, but it has the virtue of being the correct advice. Just as scientists can conduct research and extend human knowledge only by trusting well, so we laypeople must trust well. If we don’t already trust well, the criteria won’t help us trust better.

## Conclusion

A central goal of much social epistemology is not merely to understand how belief formation works, but to improve it. Social epistemology has the ambition of being *regulative* (Ballantyne, 2019a).^13^ If the view I’ve defended here is on the right track, our ambitions should be dramatically scaled back, at least with regard to the novice/expert problem. There’s little useful advice we can give to novices about how to choose trustworthy experts. They’re either already doing what they should, by their own lights – extending their trust – or they’re unable to deploy the criteria usefully. The same is true of us, of course, and for the same reasons: we’re novices too, in most of these domains. We’re all extending our trust. Mostly, we do this unthinkingly (though intelligently). Sometimes, we do it deliberately, but I doubt there are many people who need to be told to extend trust in this way (fewer still who don’t do it already and have the capacity to do it with instruction).

There *is* a regulative project for us to engage in, but it’s not a task for epistemologists alone. We live in an epistemically hostile environment; an environment in which there are multiple agents who seek to secure goals by taking advantage of our cognitive vulnerabilities (Nguyen, 2023; Stanovich, 2018). While we cannot hope to offer the sort of epistemic backstop that philosophers like Goldman and Anderson hope for, we can engineer our epistemic environment to reduce the influence of such hostile actors. We’ve faced analogous challenges before: for example, as societies became larger and more anonymous (thanks, in part, to innovations in transportation that enabled people to move relatively freely between cities and even countries), sellers and buyers could no longer rely on personal reputation to ensure quality of goods or creditworthiness. They evolved a variety of mechanisms to fill the gap, some top-down and dependent on government, some bottom-up, like trade associations and stock exchanges (Phillips and French 1998; Vernon, 2014). We urgently need epistemic equivalents.

Such epistemic mechanisms will ensure that signals for trustworthiness better align with reliability – for example, ensuring that news sources that are more reliable have a wider reach, that genuine experts are made more salient than charlatans, and that well-validated claims are endorsed by institutions that are widely trusted.^14^ We might expect such mechanisms to evolve through similar sorts of processes as in the commercial sphere, some through deliberate regulation and legislation, some more organically, some driven by ordinary agents and reliant on the wisdom of crowds and some driven by commercial interests. None of this is at all easy, of course, especially if it is to be done in a way that avoids putting inordinate power into the hands of governments, corporations, or individuals who will be inevitably tempted to abuse it, but it’s where our efforts are best invested.^15^

## Footnotes

1 There is a lively and well-developed debate on the nature of expertise (see Grundmann forthcoming; Watson, 2020 for an overview). My definition is designed to be neutral on the issues central to this debate, perhaps at the expense of generality. That’s not a cost from the point of view of the project I’m engaged in here: if there are kinds of expertise the definition excludes, they’re not the kinds to which we must defer to acquire justified beliefs.

2 Arguably, adjudicating between competing answers to a question requires less expertise than attempting to answer it from scratch. The person attempting the adjudication may, for example, build on areas of agreement between conflicting experts, and defer to that consensus about what matters are central in answering the question reliably.

3 Hardwig (1991) actually characterises the kind of trust at issue as “blind”, and Goldman follows him in this usage. I prefer to avoid terminology that might reasonably be seen as ableist.

4 I’m grateful to a reviewer for this journal for emphasising the need to talk about citations in this context.

5 Laypeople often hold an individualist view of science; that is, they think that scientists should rarely or never defer to one another. John (2018) argues that a mistaken folk psychology of science can lead laypeople to mistake routine and epistemically justified scientific practice for scientific fraud or corruption. The distributed nature of scientific cognition belongs on the list of good scientific practices that might strike novices as iffy.

6 A reviewer for this journal suggests that non-expert adjudicators are capable of assessing the nature of patterns of deference, and that doing so will aid them in distinguishing reliable experts from unreliable dissenters. The reviewer suggests that genuine experts, but not unreliable dissenters, will exhibit long-standing cross-generational connections with respect to training and citation patterns in scientific journals. I agree that such patterns distinguish reliable experts from cranks. But I am doubtful they enable us to distinguish reliable experts from sophisticated mimics of expertise. As we’ve seen, they are or are able to enlist genuinely well-credentialed, sophisticated voices. They will typically claim that it is them, and not the mainstream, who are the inheritors of the scientific tradition. They may not be able to cite as many well-credentialed people who can plausibly claim an impeccable lineage on their side, but they will point to gatekeeping and group think to explain that fact.

7 As a reviewer for this journal points out, this is a problem that lies at the core of the scientific enterprise, and at least to some degree is independent of questions of selective trust. This is all the more reason to think it’s not a criterion that’s helpful to lay observers.

8 I define epistemic trespassing in terms of adjudication - an epistemic trespasser attempts to adjudicate an issue in a domain in which they lack expertise - to avoid the kinds of problems that arise when we define it less strictly. Both Gerken (2023) and Watson (2022) define it in terms of simple boundary crossing. Their definitions yield the result that epistemic trespassing is often, perhaps usually, unproblematic. Scientific collaboration regularly involves boundary crossing, as Gerken notes, and there’s nothing impermissible in experts in one domain querying the conclusions of those in a closely related area, as Watson points out. But neither case involves adjudication. As I’m using the word, adjudication occurs when one expert comes to a conclusion about a question outside their own competence, not on the basis of testimony but on the basis of their own assessment of the evidence. Of course, one may define terms how one likes: the advantage of my definition, I claim, is that it isolates the epistemically problematic cases.

9 On some accounts, expertise is democratized, such that (almost) everyone is an expert in something(s): natural language, say, or negotiating the social world (Collins and Evans 2008). On this view, novice adjudication of issues in an expert domain just is epistemic trespassing.

10 That it has the features that make epistemic trespassing unreliable does not entail that it gives rise to all the same problems as epistemic trespassing. DiPaolo (2022) points out that expertise has a social role, and that social role raises special problems when a genuine expert trespasses, as compared to a novice. The expert may take advantage of her status to give her pronouncements unearned weight, and thereby make third parties worse off epistemically. The novice has no such status and is less likely to sway third parties and may be expected to acknowledge their lack of expertise. The risks of lay adjudication in expert domains are therefore smaller, and the layperson is correspondingly under no (or little) obligation to avoid it. None of this clears lay adjudication of its epistemic failings, however: just as the knowledge *that judgment j has been generated via epistemic trespassing* is a defeater for *j*, so the knowledge that *j** has been generated through the novice version of epistemic trespassing is a defeater for *j*.*

11 Brennan (2020) notes that it can often be difficult to know whether a putative expert is in fact an epistemic trespasser. He gives the example of William Happer, who denies that climate change is anthropogenic. Happer is a physicist, and therefore adjudged an epistemic trespasser by Brennan - “but how is a novice supposed to know that?” (230). In fact, the problem is more intractable than Brennan seems to recognize: climate science is highly interdisciplinary and calls upon the expertise of physicists as well as experts in many other domains (Hegerl, 2022). *That someone adjudicating an issue in climate science is a physicist* is not dispositive evidence that they’re an epistemic trespasser.

12 Of course, the average viewer of Fox News won’t have checked the credentials or track records of the apparent experts they hear expressing scepticism about climate change. Neither will the average reader of the *New York Times*. But they each respond rationally to the minimal cues to expertise they’re provided with, such as a caption or a spoken introduction that mentions their expertise (e.g., by citing their institution and their position). Viewers trust these sources and therefore trust that the experts they choose are reliable; typically, these news sources choose experts that their viewers would trust were they to investigate further, so viewers rationally outsource the checking.

13 McKenna (2023) prefers the term ‘inquiry epistemology’, which he credits to Alfano (2012). Inquiry epistemology has both descriptive and regulative goals. McKenna worries that the term “regulative epistemology” risks underplaying the descriptive side that any epistemology surely need.

14 This is a conclusion I share with McKenna (2023), who in turn credits it to Anderson (2011). Anderson’s diagnosis of the epistemic problem is different to mine, however, and the solution she offers is correspondingly different. Anderson argues that the sort of criteria she and Goldman offer provide reliable means of distinguishing reliable from unreliable experts right now, in the current epistemic environment. She suggests that the difficulty in identifying experts arises more from threats to the disposition to deploy these criteria, rather than from any in-principle obstacle to their reliable use. Her proposals are correspondingly focused on promoting dialogue across partisan divides and on exposure to a wide range of information. I don’t share her diagnosis of the problem (see Levy, 2021 for discussion), and my suggestions for amelioration are instead focused on the cues for reliability of information, rather than on the partisan divide.

15 I am grateful to two reviewers for *Erkenntnis* for extremely helpful comments on an earlier version of this paper. I am also grateful to the Arts and Humanities Research Council (AH/W005077/1) and the John Templeton Foundation (grant #62,631) for their generous support.
